# Supplementary material for: Aryl hydrocarbon receptor is required for optimal B‐cell proliferation
Source: EMBO J. 2016 Nov 14;36(1):116–28. doi: 10.15252/embj.201695027 (PMC5210087; doi:10.15252/embj.201695027)
Supplement: Supplementary file 1 — Appendix [file EMBJ-36-116-s001.pdf]

Appendix figures and tables

- Appendix figure S1. *Ahr*$^{-/-}$ mice show altered steady-state production of serum antibodies.
- Appendix figure S2. AhR deficiency does not affect survival of B cells.
- Appendix figure S3. AhR deficiency affects the number of steady-state splenic plasma cells but not steady-state bone marrow-resident plasma cells or immunization-derived plasma cells.
- Appendix figure S4. *Ahr* deletion does not alter p27kip1 expression, up-regulation of activation markers or calcium mobilization in B cells.
- Appendix figure S5. *Ccno* gene contains multiple Dioxin Response Elements (DREs).

- Appendix table S1. RNAseq top 20 down-regulated genes in *Ahr*$^{-/-}$ B cells.
- Appendix table S2. RNAseq top 20 up-regulated genes in *Ahr*$^{-/-}$ B cells.

Appendix figure S1

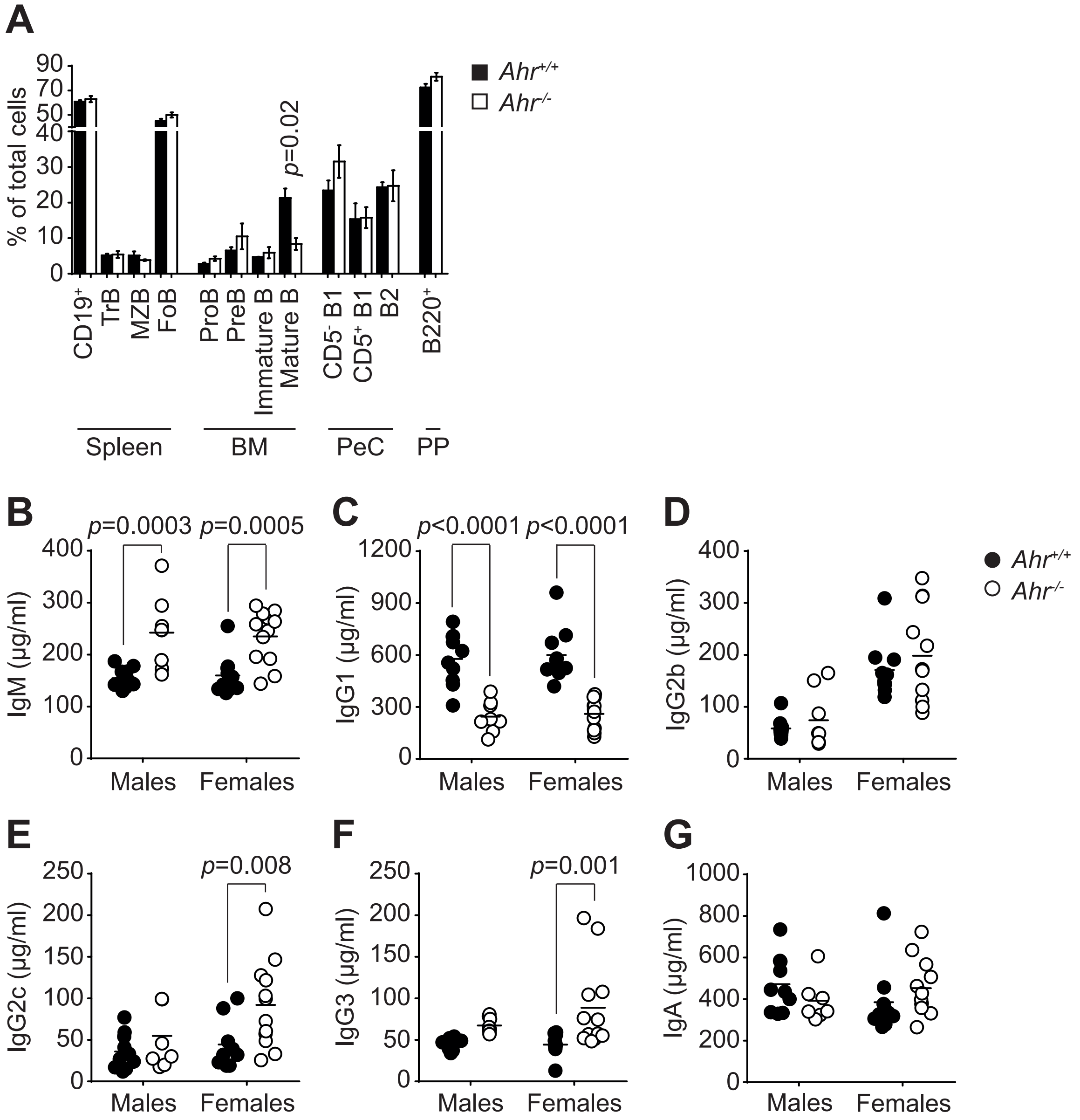

Appendix figure S1. ***Ahr*$^{-/-}$ *mice* show altered steady-state production of serum antibodies.**

A Flow cytometry analysis of distribution of B cell subsets sorted from spleen, bone marrow (BM), peritoneal cavity (PeC) and Peyer's patches (PP) of 8 weeks-old male non-immune *Ahr*$^{+/+}$ (black) and *Ahr*$^{-/-}$ (white) mice. n = 3 mice per group; mean ± SEM; unpaired two-tailed *t* test.

B-G ELISA quantification of indicated antibody isotypes in the serum of 8 weeks-old male and female non-immune *Ahr*$^{+/+}$ (black) and *Ahr*$^{-/-}$ (white) mice. Line indicates mean value; unpaired two-tailed *t* test.

Appendix figure S2

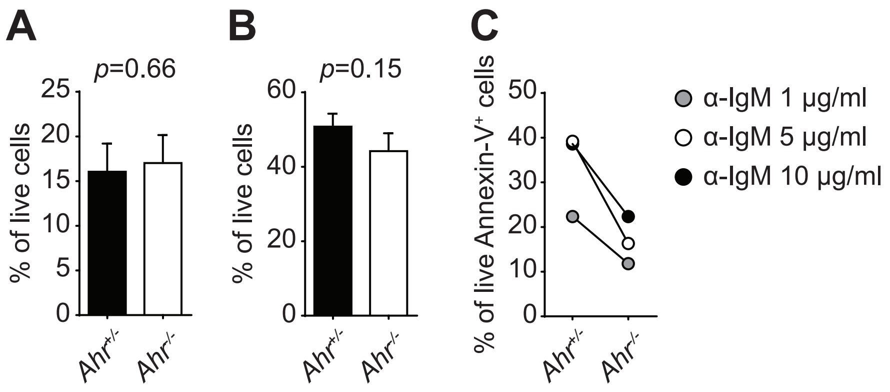

Appendix figure S2. AhR deficiency does not affect survival of B cells.

A Flow cytometry analysis of splenic $CD19^+$ cells isolated from non-immune *Ahr*$^{fl/+}$*mb1*$^{Cre+}$ (black) and *Ahr*$^{fl/-}$*mb1*$^{Cre+}$ (white) mice and cultured for 72h in medium alone. Data from n = 4 independent experiments. Paired two-tailed *t* test.

B Flow cytometry analysis of splenic $CD19^+$ cells isolated from non-immune *Ahr*$^{fl/+}$*mb1*$^{Cre+}$ (black) and *Ahr*$^{fl/-}$*mb1*$^{Cre+}$ (white) mice and cultured for 72h with 20 ng/ml IL-4. Data from n = 4 independent experiments. Paired two-tailed *t* test.

C Flow cytometry analysis of splenic $CD19^+$ cells isolated from non-immune *Ahr*$^{fl/+}$*mb1*$^{Cre+}$ and *Ahr*$^{fl/-}$*mb1*$^{Cre+}$ mice and cultured for 24h with the indicated stimuli. Data from n = 1 experiment.

Appendix figure S3

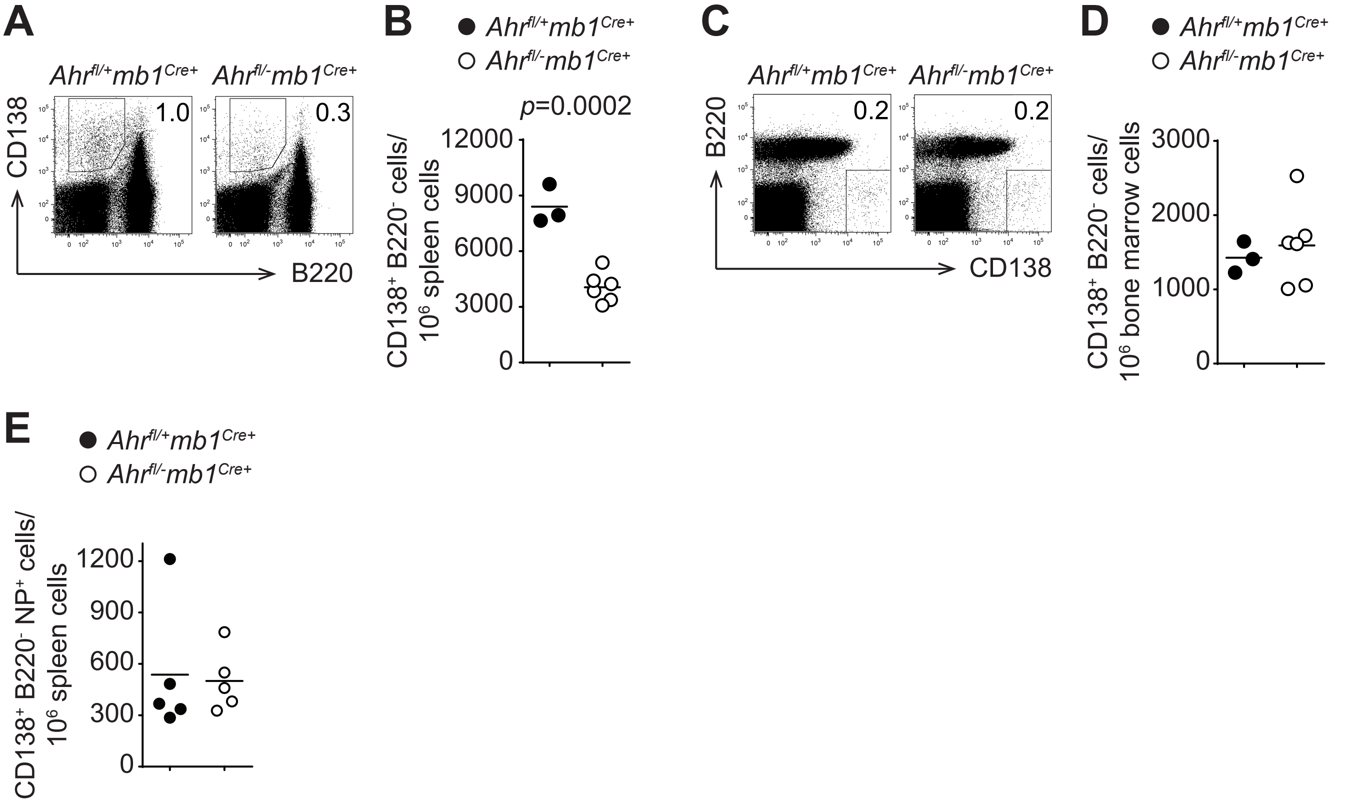

Appendix figure S3. AhR deficiency affects the number of steady-state splenic plasma cells but not steady-state bone marrow-resident plasma cells or immunization-derived plasma cells.

A, **C** Flow cytometry analysis of distribution of splenic (A) and bone marrow (C) B220$^+$ CD138$^+$ plasma cells in non-immune *Ahr*$^{fl/+}$*mb1*$^{Cre+}$ (black) and *Ahr*$^{fl/-}$*mb1*$^{Cre+}$ (white) mice. Representative data of n = 2 independent experiments.

B, **D** Number of plasma cells per 10^6 total cells in spleen (B) and bone marrow (D), as gated in A and C. n = 2 independent experiments; lines indicate mean; unpaired two-tailed *t* test.

E Number of NP-specific plasma cells per 10^6 total cells in spleen of *Ahr*$^{fl/+}$*mb1*$^{Cre+}$ (black) and *Ahr*$^{fl/-}$*mb1*$^{Cre+}$ (white) mice immunized i.p. with 10 µg/mouse of the T-dependent model antigen NP-CGG, 7 days post-challenge. n = 1 experiment; lines indicate mean.

Appendix figure S4

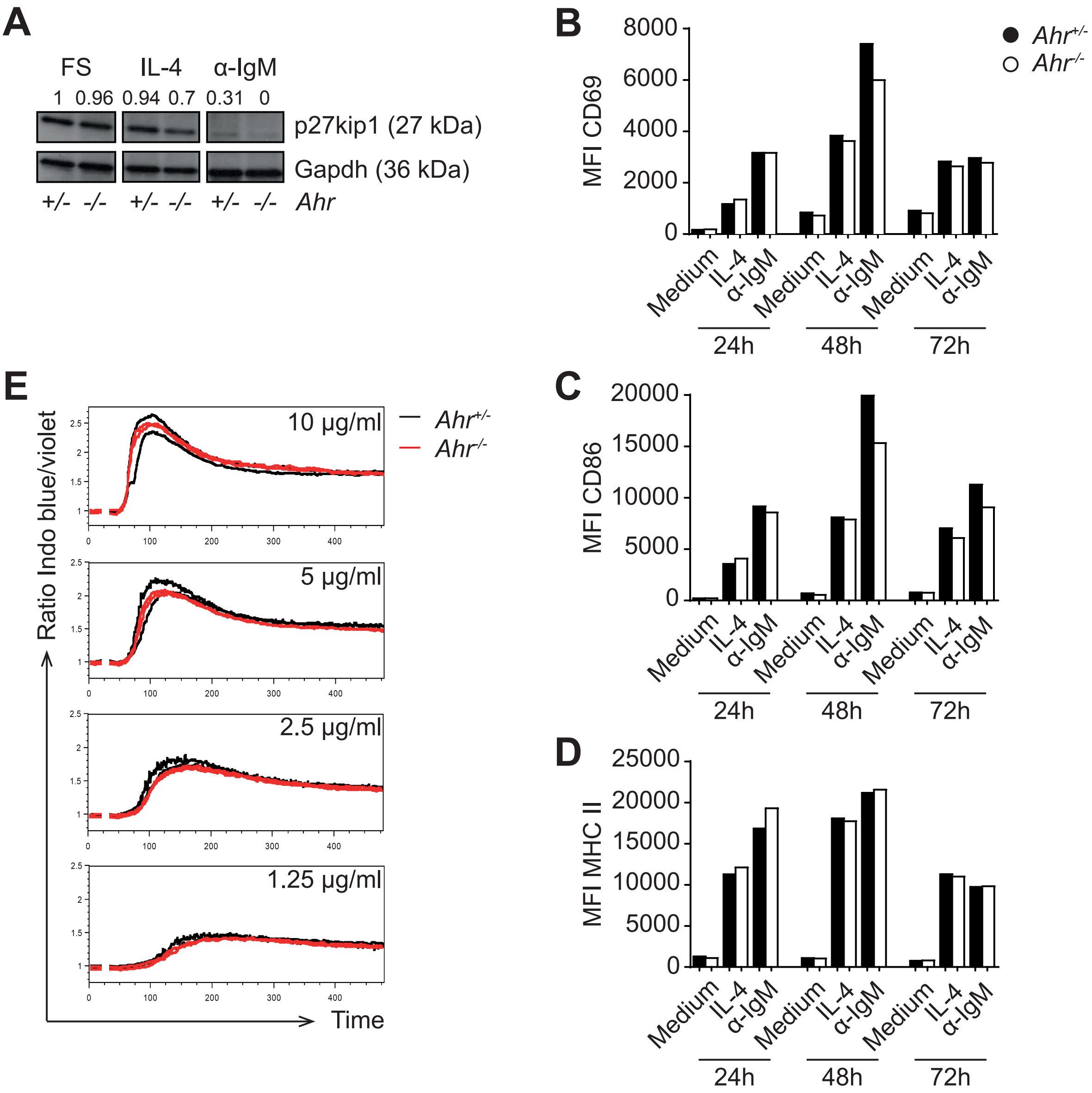

Appendix figure S4. *Ahr* deletion does not alter p27kip1 expression, up-regulation of activation markers or calcium mobilization in B cells.

A Western blot of whole protein extract from splenic $CD19^+$ cells isolated from non-immune $Ahr^{fl/+}mb1^{Cre+}$ ($Ahr^{+/-}$ B cells) and $Ahr^{fl/-}mb1^{Cre+}$ ($Ahr^{-/-}$ B cells) mice and cultured for 24h as indicated (α-IgM 5 μg/ml). Values above the blots indicate p27kip1 protein quantification obtained by densitometry, normalized to Gapdh and compared to FS $Ahr^{fl/+}mb1^{Cre+}$ sample. Data from n = 1 experiment. FS: freshly sorted.

B-D Flow cytometry analysis of CD69 (B), CD86 (C) and MHC II (D) surface expression, indicated as mean fluorescence intensity (MFI), on splenic $CD19^+$ cells isolated from non-immune $Ahr^{fl/+}mb1^{Cre+}$ (black) and $Ahr^{fl/-}mb1^{Cre+}$ (white) mice and cultured as indicated (α-IgM 5 μg/ml). Data from n = 1 experiment.

E Flow cytometry analysis of calcium flux (indicated as ratio Indo blue/violet) over time of FoB cells isolated from non-immune $Ahr^{fl/+}mb1^{Cre+}$ (black) and $Ahr^{fl/-}mb1^{Cre+}$ (red) mice and stimulated with α-IgM at the indicated concentrations. Representative data of n = 2 independent experiments.

Appendix figure S5

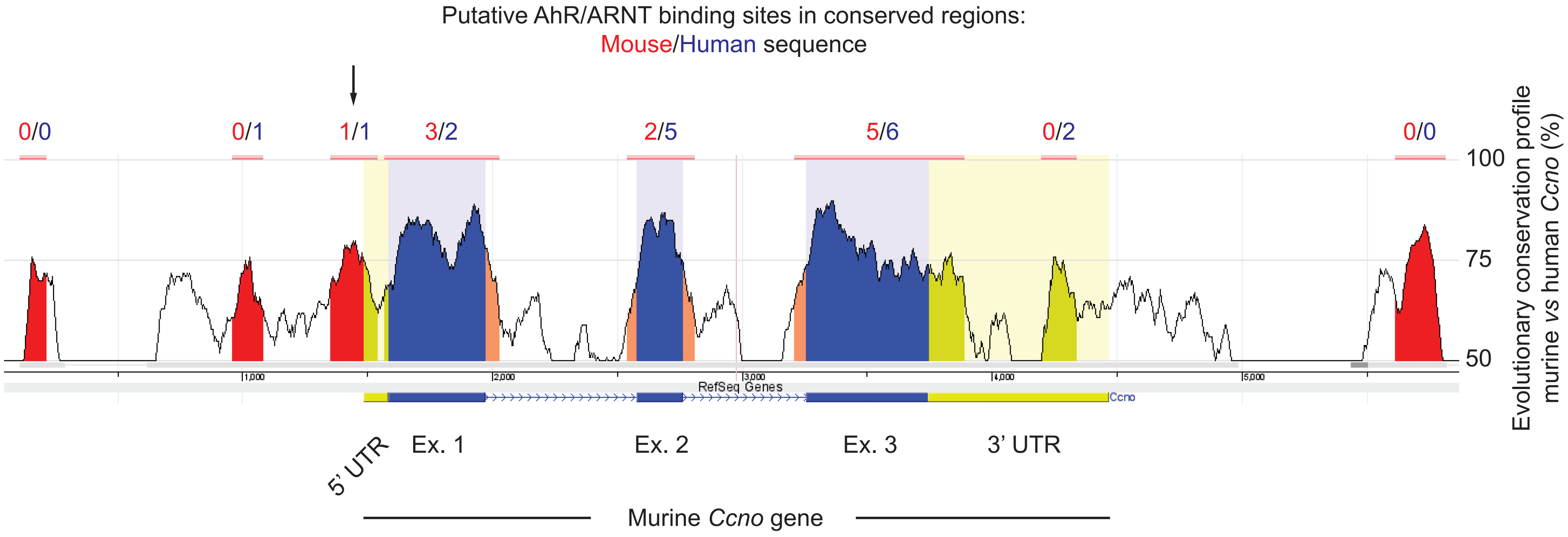

Appendix figure S5. *Ccno* gene contains multiple Dioxin Response Elements (DREs).
Conservation analysis was performed between mouse and human *Ccno* sequences to identify conserved regions that are likely to contain putative DREs. Analysis showed that several regions within the *Ccno* locus contains conserved putative DREs. A putative conserved DRE was identified in proximity to the 5' UTR. This DRE is localized into the region bound by AhR, as showed in Figure 6F. Threshold for sequence similarity: 75%. Threshold for AhR/ARNT binding site sequence similarity: 80%. Analysis performed on ECR Browser.

Appendix table S1

Gene symbol	*ENSEMBL gene ID*	*Description*	*Function*	*Avg read count Ahr^+/+^*	*Avg read count Ahr^-/-^*	*Fold change Ahr^+/+^ vs Ahr^-/-^*	*Adjusted p value*
Cyp1b1	ENSMUSG00000024087	Cytochrome P450 1B1	Detoxification	207	0	- infinite	7.23×10^{-19}
Myl10	ENSMUSG00000005474	Myosin regulatory light chain 10	Regulation of actin cytoskeleton	8	0	- infinite	0.00236
Tmprss4	ENSMUSG00000032091	Transmembrane protease serine 4	Serine protease	9	0	- infinite	2.15×10^{-5}
Cyp1a1	ENSMUSG00000032315	Cytochrome P450 1A1	Detoxification	4733	5	-979	3.34×10^{-114}
Ahrr	ENSMUSG00000021575	Aryl hydrocarbon receptor repressor	Transcriptional repressor of AhR pathway	297	2	-178	1.85×10^{-74}
Ccno	ENSMUSG00000042417	Cyclin-O	Cell cycle; multiciliogenesis	94	1	-119	2.95×10^{-42}
Sema3b	ENSMUSG00000057969	Semaphorin 3B	Neuronal development	44	1	-58	2.82×10^{-15}
Rtn4rl2	ENSMUSG00000050896	Reticulon 4 receptor-like 2	Neuronal development	12	0	-56	0.00043
Fhod3	ENSMUSG00000034295	Formin homology 2 domain containing 3	Regulation of actin cytoskeleton	14	0	-52	0.00196
Mpp2	ENSMUSG00000017314	MAGUK p55 subfamily member 2	Cytoskeleton regulation	32	1	-50	3.39×10^{-13}
Asb2	ENSMUSG00000021200	Ankyrin repeat and SOCS box protein 2	Protein degradation	334	8	-44	7.1×10^{-12}
Gm15880	ENSMUSG00000084821	Predicted gene 15880		9	0	-42	0.00019
1700030C10Rik	ENSMUSG00000099759	RIKEN cDNA 1700030C10 gene		33	1	-39	1.71×10^{-12}
Ovol1	ENSMUSG00000024922	Putative transcription factor Ovo-like 1	Transcription factor	22	1	-31	2.23×10^{-5}
Muc19	ENSMUSG00000044021	Mucin 19	Glycoprotein involved in mucus formation	14	1	-25	3.71×10^{-5}
Hic1	ENSMUSG00000043099	Hypermethylated in cancer 1 protein	Tumor repressor; transcriptional repressor	2293	98	-23	0
Nqo1	ENSMUSG00000003849	NAD(P)H quinone dehydrogenase 1	Reductase	1102	49	-23	6.29×10^{-266}
Pltp	ENSMUSG00000017754	Phospholipid transfer protein	Lipid metabolism	1277	59	-22	7.49×10^{-172}
Tiparp	ENSMUSG00000034640	TCDD-inducible poly(ADP-Ribose) polymerase	Poly(ADP-ribose) polymerase	21667	1098	-20	0
Bfsp1	ENSMUSG00000027420	Beaded filament structural protein 1	Structural protein	18	1	-19	1.51×10^{-5}

Appendix table S1. RNAseq top 20 down-regulated genes in *Ahr$^{-/-}$* B cells.
CD19^{+} cells were isolated from *Ahr$^{+/+}$* and *Ahr$^{-/-}$* mice and cultured for 4h in presence of 10 µg/ml α-IgM and 20 ng/ml IL-4, followed by additional 4h in presence of 250 nM FICZ. Genes were ranked according to decreasing fold change difference between *Ahr$^{+/+}$* and *Ahr$^{-/-}$* B cells. Gene symbol was assigned according to the MGI database. Average (avg) read counts indicate the number of reads per gene and are directly proportional to the extent of gene expression.

Appendix table S2

Gene symbol	ENSEMBL gene ID	Description	Function	Avg read count $Ahr^{+/+}$	Avg read count $Ahr^{-/-}$	Fold change $Ahr^{+/+}$ vs $Ahr^{-/-}$	Adjusted p value
Kifc3	ENSMUSG00000031788	Kinesin family member C3	Cell cycle	62	257	4	3.22×10^{-23}
Chdh	ENSMUSG00000015970	Choline dehydrogenase	Lipid/neurotransmitter metabolism	6	27	4	5.46×10^{-5}
Fam83f	ENSMUSG00000022408	Family with sequence similarity 83, member F		37	159	4	4.08×10^{-15}
Gpr35	ENSMUSG00000026271	G protein-coupled receptor 35	Receptor for kynurenic acid	8	37	4	8.83×10^{-4}
Mcam	ENSMUSG00000032135	Melanoma cell adhesion molecule	Cell ashesion	7	32	4	7.05×10^{-5}
Stard13	ENSMUSG00000016128	StAR-related lipid transfer domain containing 13	Cytoskeleton regulation	3	16	5	8.17×10^{-4}
Klk1	ENSMUSG00000063903	Kallikrein 1	Serine protease	8	37	5	8.62×10^{-7}
Siglech	ENSMUSG00000051504	sialic acid binding Ig-like lectin H	Immune inhibitory receptor	51	243	5	5.6×10^{-39}
Lair1	ENSMUSG00000055541	Leukocyte-associated Ig-like receptor 1	Immune inhibitory receptor	11	55	5	1.34×10^{-6}
Fabp4	ENSMUSG00000062515	Fatty acid binding protein 4	Lipid transport protein	5	26	5	6.11×10^{-4}
3830403N18Rik	ENSMUSG00000031125	RIKEN cDNA 3830403N18 gene		6	32	5	4.78×10^{-6}
Irs1	ENSMUSG00000055980	Insulin receptor substrate 1	Insulin receptor pathway	13	64	5	1.47×10^{-15}
Npl	ENSMUSG00000042684	N-acetylneuraminate pyruvate lyase	Metabolism of sialic acid	2	13	5	6.09×10^{-3}
Cd209a	ENSMUSG00000031494	CD209a antigen (DC-SIGN)	Pattern recognition receptor	6	33	6	4.7×10^{-5}
Ccl17	ENSMUSG00000031780	Chemokine (C-C motif) ligand 17	Chemokine effective on T cells; binds CCR4	7	47	6	6.02×10^{-7}
Tmem221	ENSMUSG00000043664	Transmembrane protein 221		2	11	7	6.16×10^{-4}
Gm15645	ENSMUSG00000086414	Predicted gene 15645		81	1048	13	5.19×10^{-190}
Cd209d	ENSMUSG00000031495	CD209d antigen (SIGNR3)	Pattern recognition receptor	1	16	13	4.42×10^{-7}
4930447C04Rik	ENSMUSG00000021098	RIKEN cDNA 4930447C04 gene		2	34	16	5.27×10^{-7}
Pxdn	ENSMUSG00000020674	peroxidasin	Extracellular matrix formation	0	5	Infinite	8.21×10^{-3}

Appendix table S2. RNAseq top 20 up-regulated genes in *$Ahr^{-/-}$* B cells.
$CD19^{+}$ cells were isolated from *$Ahr^{+/+}$* and *$Ahr^{-/-}$* mice and cultured for 4h in presence of 10 μg/ml α-IgM and 20 ng/ml IL-4, followed by additional 4h in presence of 250 nM FICZ. Genes were ranked according to increasing fold change difference between *$Ahr^{+/+}$* and *$Ahr^{-/-}$* B cells. Gene symbol was assigned according to the MGI database. Average (avg) read counts indicate the number of reads per gene and are directly proportional to the extent of gene expression.
