# Supplementary material for: Aryl hydrocarbon receptor is required for optimal B‐cell proliferation
Source: EMBO J. 2016 Nov 14;36(1):116–28. doi: 10.15252/embj.201695027 (PMC5210087; doi:10.15252/embj.201695027)
Supplement: Supplementary file 2 — Expanded View Figures PDF [file EMBJ-36-116-s002.pdf]

Expanded View Figures

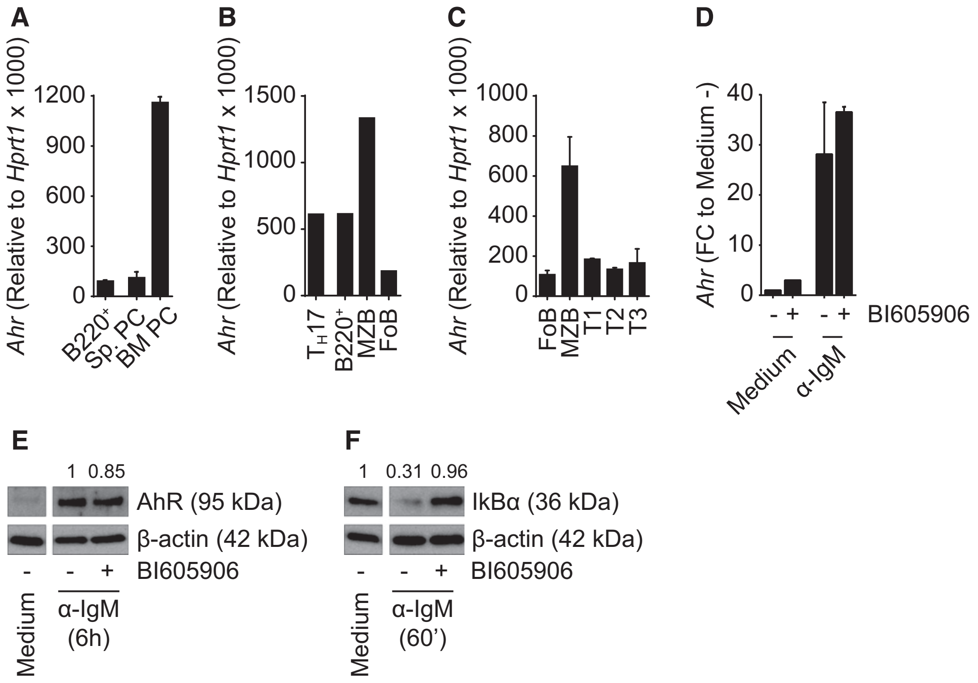

Figure EV1. AhR expression is independent of NF-κB activation.

A qPCR analysis of *Ahr* expression in splenic B220+ and plasma cell (PC) subsets and bone marrow PC subset sorted from C57Bl/6 mice. *Ahr* expression was normalized to *Hprt1*. $n = 2$ independent experiments; mean ± range.

B qPCR analysis of *Ahr* expression in T_H17 and splenic B-cell subsets sorted from *Il17Cre R26R eYFP* mice. *Ahr* expression was normalized to *Hprt1*. $n = 1$ experiment; mean.

C qPCR analysis of *Ahr* expression in splenic B-cell subsets sorted from C57Bl/6 mice. *Ahr* expression was normalized to *Hprt1*. $n = 2$ independent experiments; mean ± range.

D qPCR analysis of *Ahr* expression in splenic CD19+ cells isolated from C57Bl/6 mice and cultured for 6 h as indicated. *Ahr* expression was normalized to *Hprt1*; *Ahr* expression was normalized among groups to medium without BI605906 (medium −). $n = 2$ independent experiments; mean ± range. FC: fold change.

E Western blot analysis of whole protein extract from splenic CD19+ cells isolated from C57Bl/6 mice and cultured for 6 h as indicated. Values above the blots indicate AhR protein quantification obtained by densitometry, normalized to β-actin and compared to the sample treated with α-IgM without BI605906. Representative data of $n = 2$ independent experiments.

F Western blot analysis of whole protein extract from splenic CD19+ cells isolated from C57Bl/6 mice and cultured for 60 min as indicated. Values above the picture indicate IκBα protein quantification obtained by densitometry, normalized to β-actin and compared to the sample treated with medium without BI605906. Representative data of $n = 2$ independent experiments.

 © 2016 The Authors

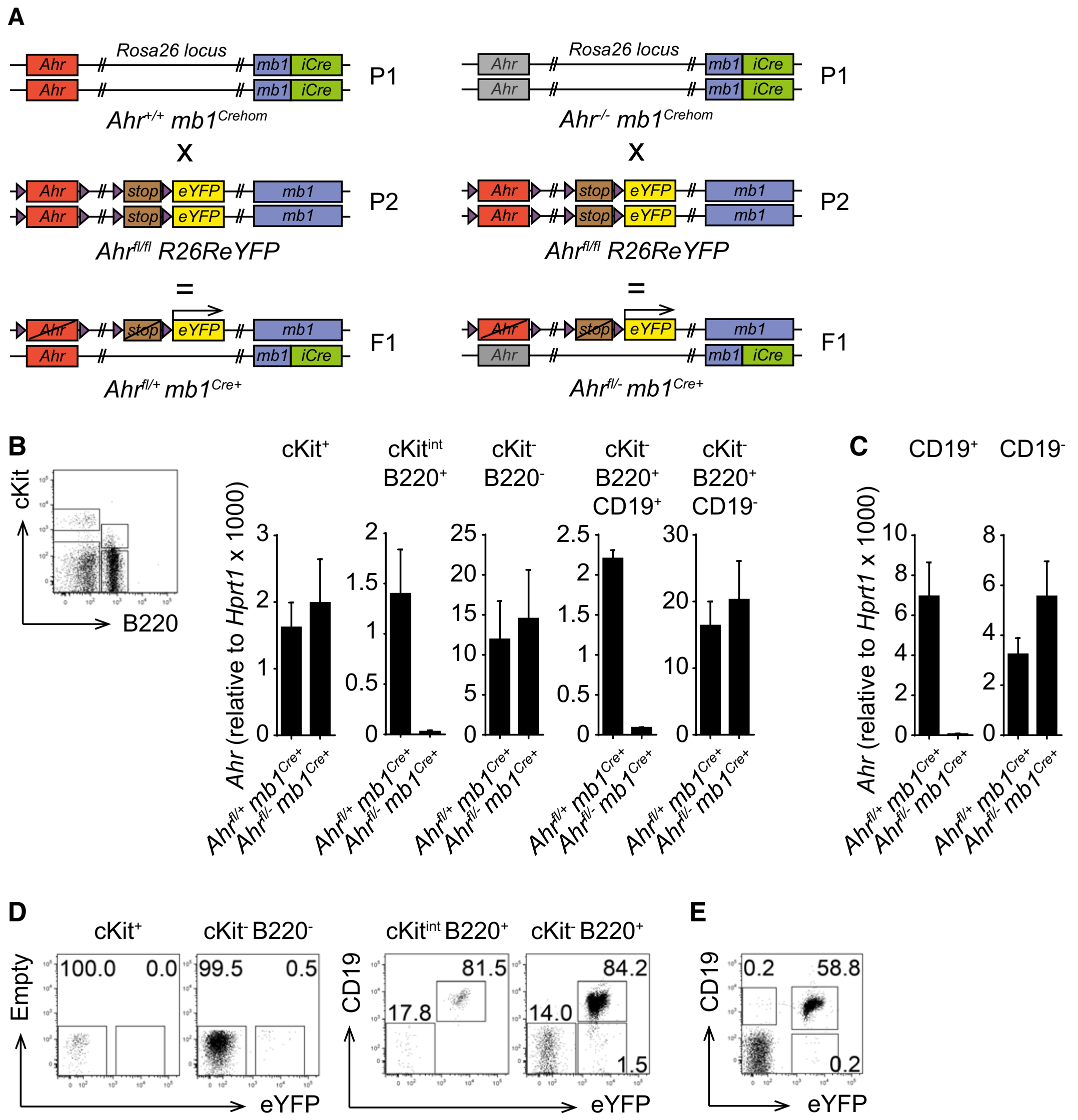

Figure EV2. The $Ahr^{fl/fl}$ *R26R eYFP* allele combined with the $mb1^{Cre}$ system allows B cell-specific *Ahr* deletion and eYFP expression.

A Breeding strategy to generate B cell-specific $Ahr^{-/-}$ mice, all carrying Cre recombinase. $Ahr^{fl/-}$ $mb1^{Cre+}$ mice lack *Ahr* in B cells. $Ahr^{fl/+}$ $mb1^{Cre+}$ mice are $Ahr^{+/-}$ in B cells. Cre activity is reported via eYFP expression.

B, C qPCR analysis of *Ahr* expression in the indicated cell subsets sorted from bone marrow (B) and spleen (C) of non-immune $Ahr^{fl/+}$ $mb1^{Cre+}$ and $Ahr^{fl/-}$ $mb1^{Cre+}$ mice. *Ahr* expression was normalized to *Hprt1*. Sorting strategy from bone marrow is depicted in the dot plot shown in (B). $n = 3$ independent experiments; mean ± SEM.

D, E Flow cytometry analysis of eYFP expression in bone marrow (D) and spleen (E) from non-immune $Ahr^{fl/+}$ $mb1^{Cre+}$ mice. Cells were gated as indicated above the dot plots. Representative data of $n = 3$ independent experiments.

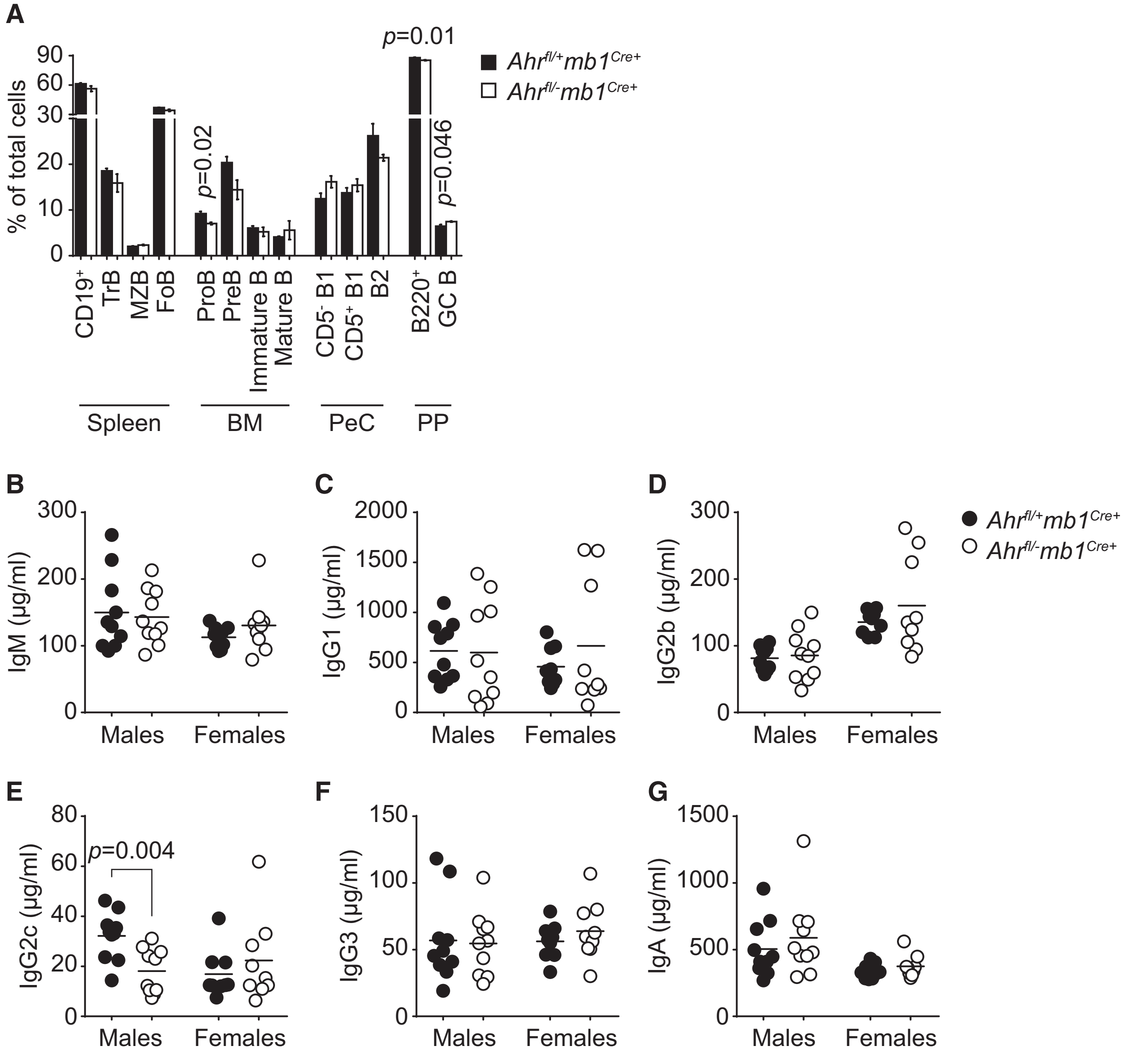

Figure EV3. B cell-specific *Ahr* deficiency does not cause overt alterations in steady-state B-cell immunity.

A Flow cytometry analysis of distribution of B-cell subsets sorted from spleen, bone marrow (BM), peritoneal cavity (PeC) and Peyer's patches (PP) of eight-week-old male non-immune $Ahr^{fl/+}$ $mb1^{Cre+}$ (black) and $Ahr^{fl/-}$ $mb1^{Cre+}$ (white) mice. n = 3 mice per group; mean ± SEM; unpaired two-tailed t-test.

B–G ELISA quantification of indicated antibody isotypes in the serum of 8-week-old male and female non-immune $Ahr^{fl/+}$ $mb1^{Cre+}$ (black) and $Ahr^{fl/-}$ $mb1^{Cre+}$ (white) mice. Line indicates mean value; unpaired two-tailed t-test.

 © 2016 The Authors

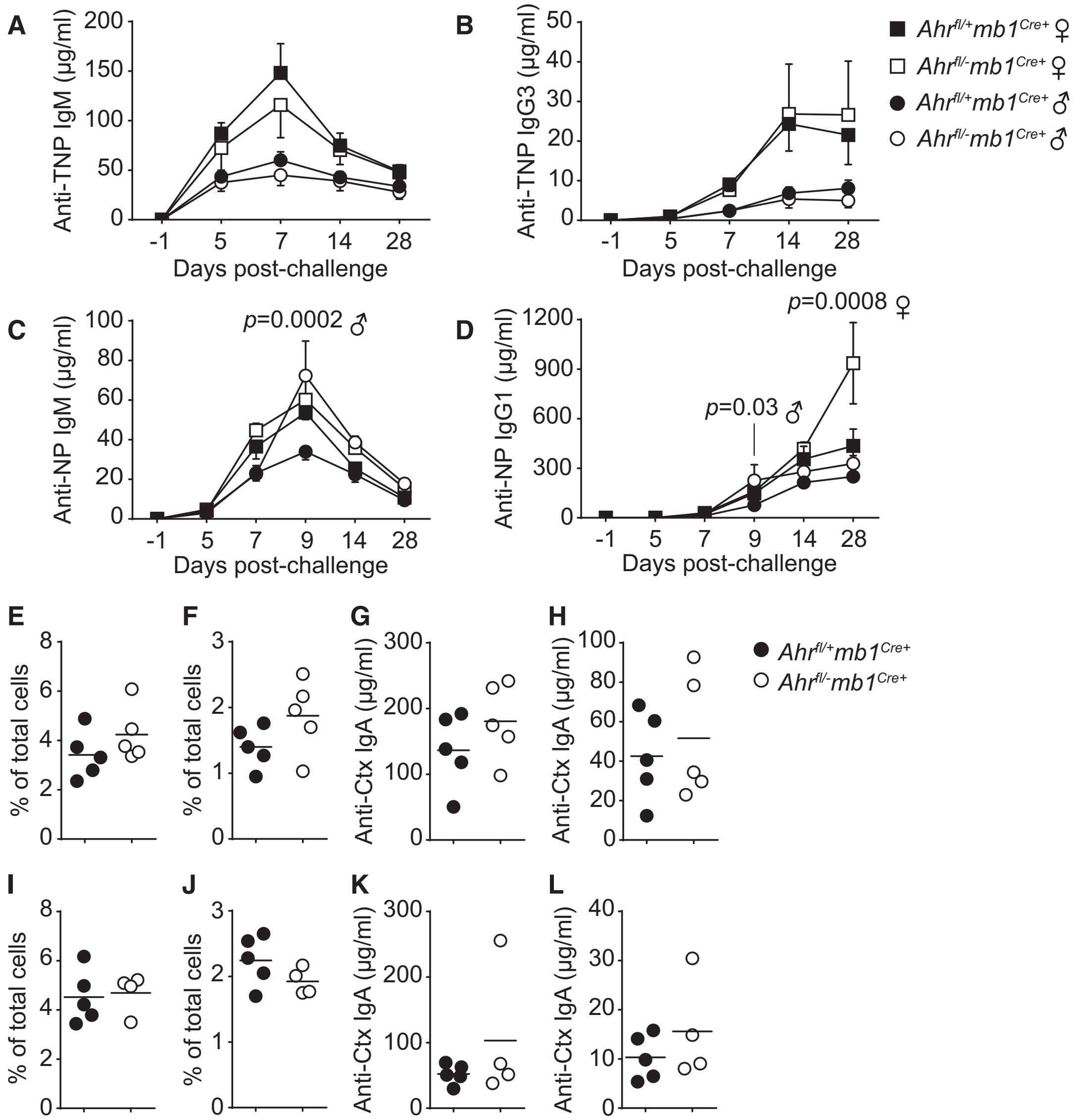

Figure EV4. B cell-specific *Ahr*-deficient mice respond normally to the T-independent antigen TNP-Ficoll, the T-dependent antigen NP-CGG and mucosal challenge with cholera toxin.

A–D ELISA quantification at indicated time points of anti-TNP IgM (A), anti-TNP IgG3 (B), anti-NP IgM (C) and anti-NP IgG1 (D) antibodies in the serum of male (circle) and female (square) $Ahr^{fl/+}$ $mb1^{Cre+}$ (black) and $Ahr^{fl/-}$ $mb1^{Cre+}$ (white) mice immunized i.p. with 10 μg/mouse TNP-Ficoll (A, B) or 10 μg/mouse NP-CGG (C, D). $n = 2$ independent experiments, five mice per group; mean ± SEM; two-way ANOVA, Sidak's test.

E–H Flow cytometry analysis of GC B-cell (E, G) and T_{FH}-cell (F, H) distributions in Peyer's patches isolated at d14 post-immunization from male (E, F) and female (G, H) $Ahr^{fl/+}$ $mb1^{Cre+}$ (black) and $Ahr^{fl/-}$ $mb1^{Cre+}$ (white) mice immunized i.g. with 2.5 μg/mouse cholera toxin (Ctx). Representative data of $n = 3$ independent experiments. Line indicates mean value.

I–L ELISA quantification at d14 post-immunization of serum (I, K) and faecal (J, L) anti-Ctx IgA antibodies from male (I, J) and female (K, L) $Ahr^{fl/+}$ $mb1^{Cre+}$ (black) and $Ahr^{fl/-}$ $mb1^{Cre+}$ (white) mice immunized i.g. with 2.5 μg/mouse cholera toxin. Representative data of $n = 3$ independent experiments. Line indicates mean value.

© 2016 The Authors

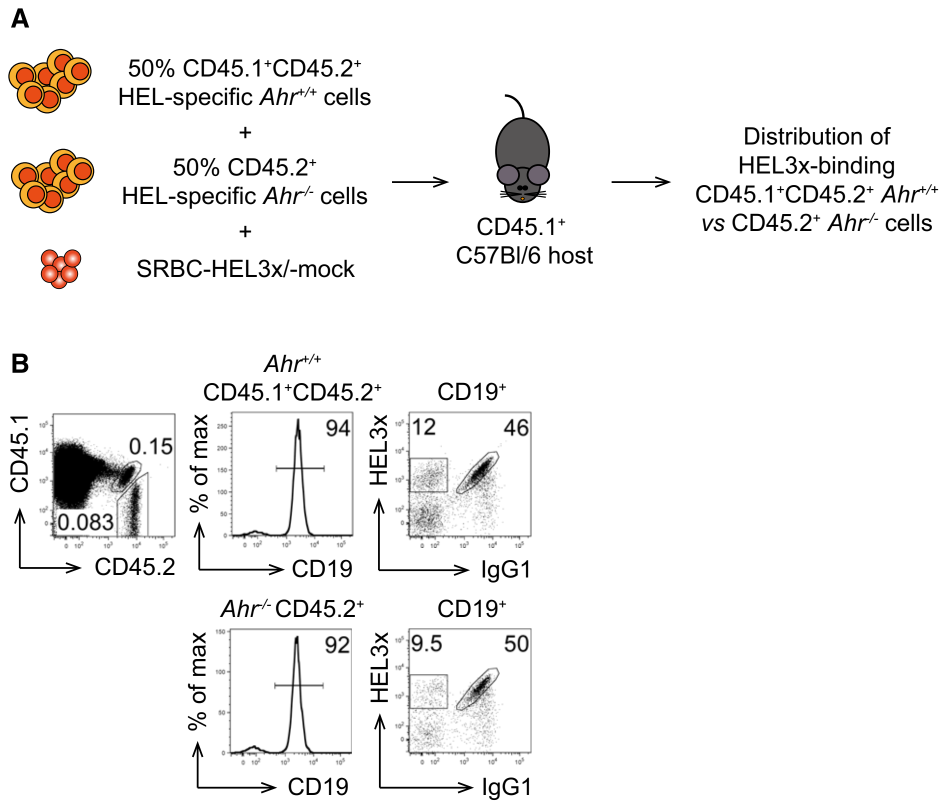

Figure EV5. AhR deficiency does not affect the affinity maturation process *in vivo*.

A Host CD45.1 mice were co-transferred with a 1:1 mixture of HEL-specific *Ahr*$^{+/+}$ CD45.1^{+}CD45.2^{+} splenocytes isolated from SW_{HEL} *Ahr*$^{+/+}$ mice and HEL-specific *Ahr*$^{-/-}$ CD45.2^{+} splenocytes isolated from SW_{HEL} *Ahr*$^{-/-}$ mice, and SRBC-HEL3x or SRBC-mock. Readout at d10 post-challenge was distribution of HEL3x-binding *Ahr*$^{+/+}$ CD45.1^{+}CD45.2^{+} vs. *Ahr*$^{-/-}$ CD45.2^{+} cells.

B Flow cytometry analysis of distribution of HEL3x-binding CD45.1^{+}CD45.2^{+} and CD45.2^{+} cells harvested from host mice challenged as indicated in (A). Cells were gated as indicated above the plots. Representative data of $n = 2$ independent experiments.

 © 2016 The Authors
